# Supplementary figures and images for: Transcription of ncRNAs promotes repair of UV induced DNA lesions in Saccharomyces cerevisiae subtelomeres
Source: PLoS Genet. 2022 Apr 29;18(4):e1010167. doi: 10.1371/journal.pgen.1010167 (PMC9106180; doi:10.1371/journal.pgen.1010167)

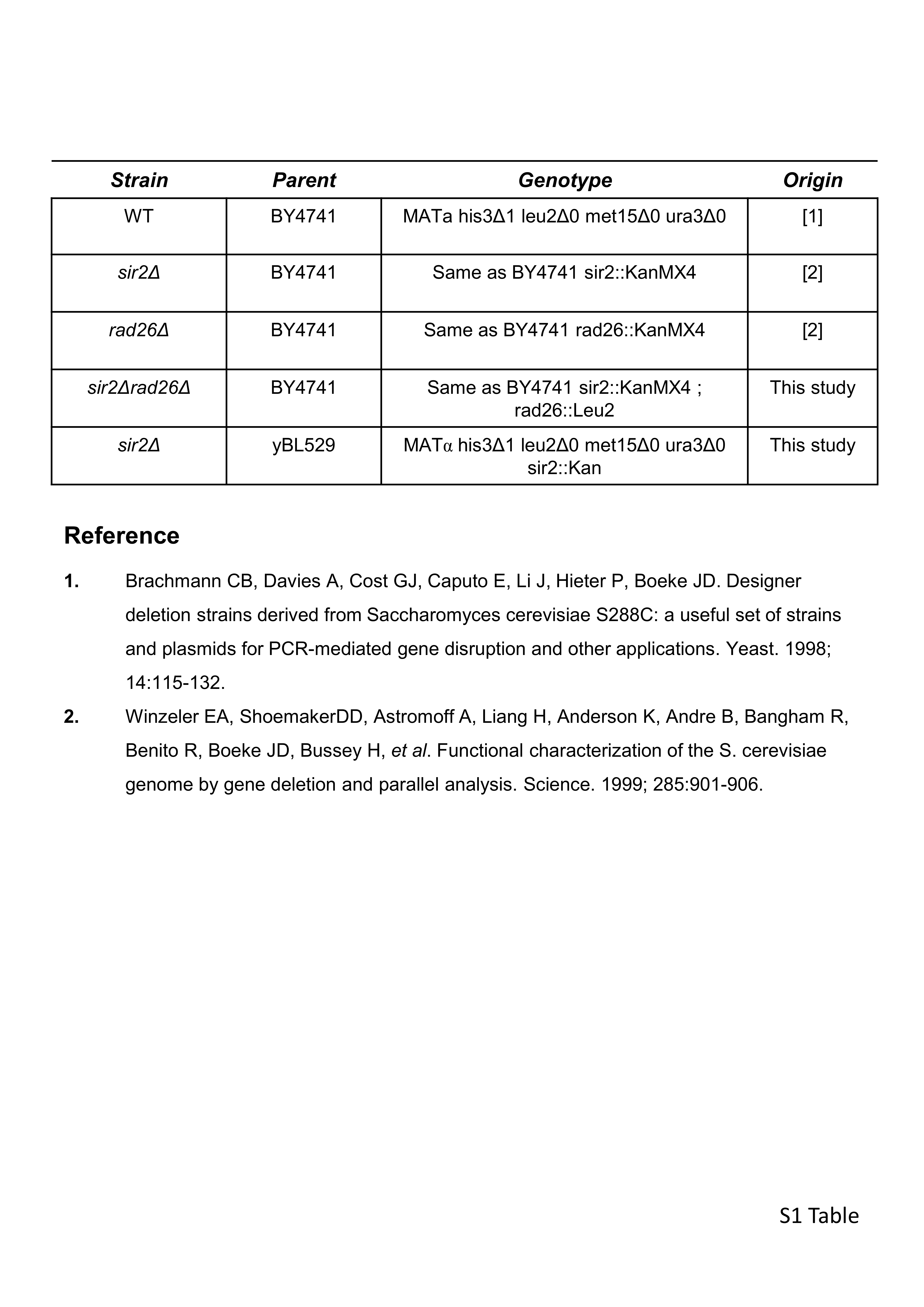

Supplement: S1 Table — (TIF) [file pgen.1010167.s001.tif]

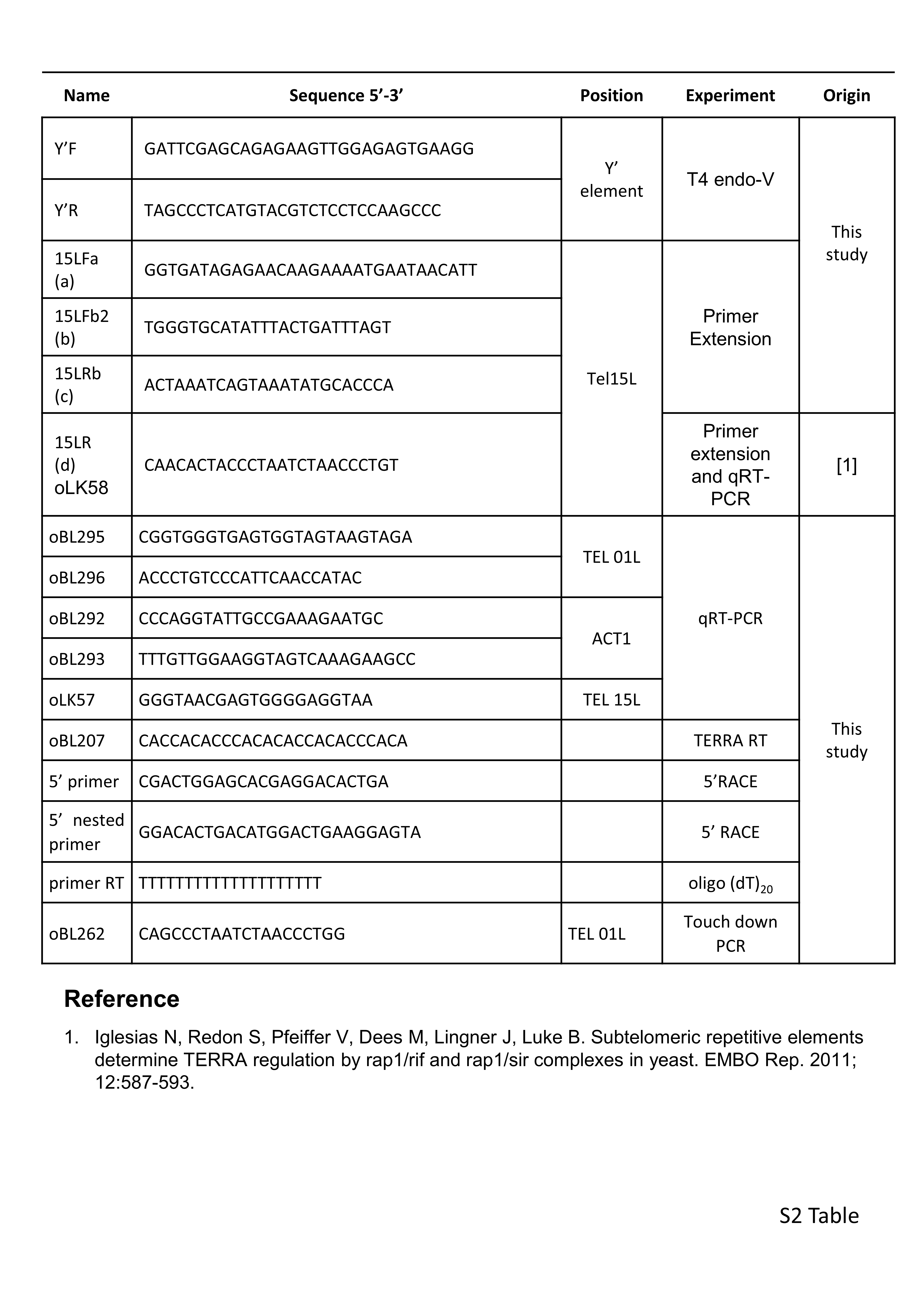

Supplement: S2 Table — Y’-element forward (Y’F) and reverse (Y’R) primers were used as 32P end-labeled single strand DNA probes (Fig 1A). Four X-element primers (‘a’ to ‘d’) were used in the Taq polymerase primer extension assay (Fig 1B). Because of high sequence similarity between repeat elements present at chromosome ends, only few unique primers were found to be specific for the X element at telomere 15L. Oligo nucleotides that were used for the 5’ RACE are shown. (TIF) [file pgen.1010167.s002.tif]

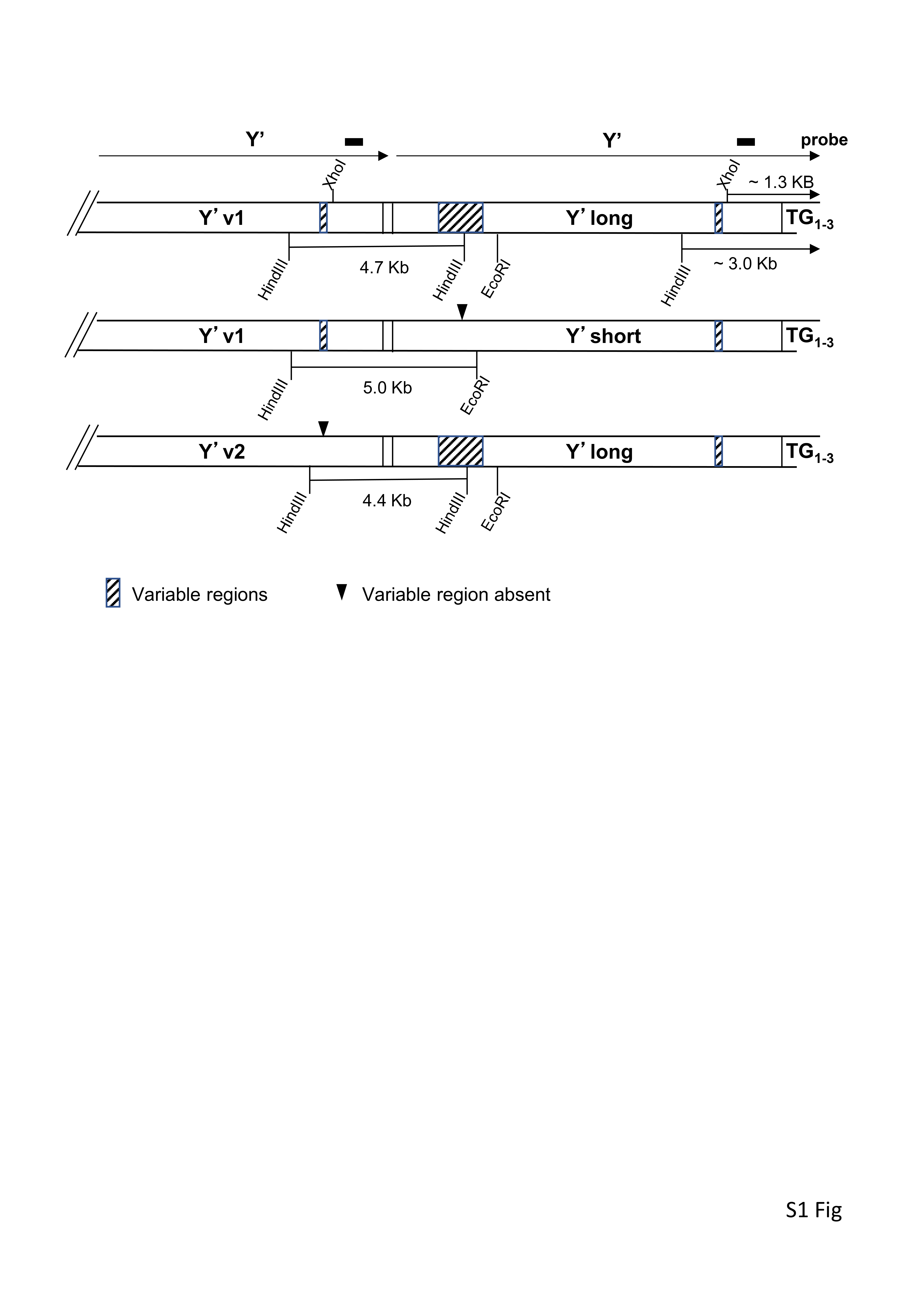

Supplement: S1 Fig — In common lab strains, about 50% of telomeres have one copy of a Y’ element telomere proximal and about 30% of those have multiple copies. When two or more Y’-elements occur on a telomere, they always have the same orientation (arrows on top). Although highly homologous, there are two areas of variability amongst Y’ elements, one of them of 1.5 kb (large striped box) and a smaller one of about 0.3 kb (small striped box). The presence or absence of the large area is denoted as Y’-long (~ 6.7 kb) and Y’-short (~ 5.2 kb) respectively. Presence or absence of the small area is not annotated but indicated as v1 and v2 in the drawing. Presence or absence of these variable elements causes occurrence of multiple restriction enzyme fragment sizes for the overlap fragment, as indicated. For the yeast used in this study, HindIII and EcoRI double digestion resulted in three overlap DNA fragments with similar lengths (4.7 kb, 5.0 kb and 4.4 kb). Note that a Y’ v2 –Y’-short combination does not exist in the strains analyzed here. The HindIII digestion also released a ~ 3.0 kb terminal fragment covering the telomere, and XhoI released a terminal fragment of ~ 1.3 kb. Because of the variable length of the telomeric repeats, these latter terminal fragments appear smeary on gels. (TIF) [file pgen.1010167.s003.tif]

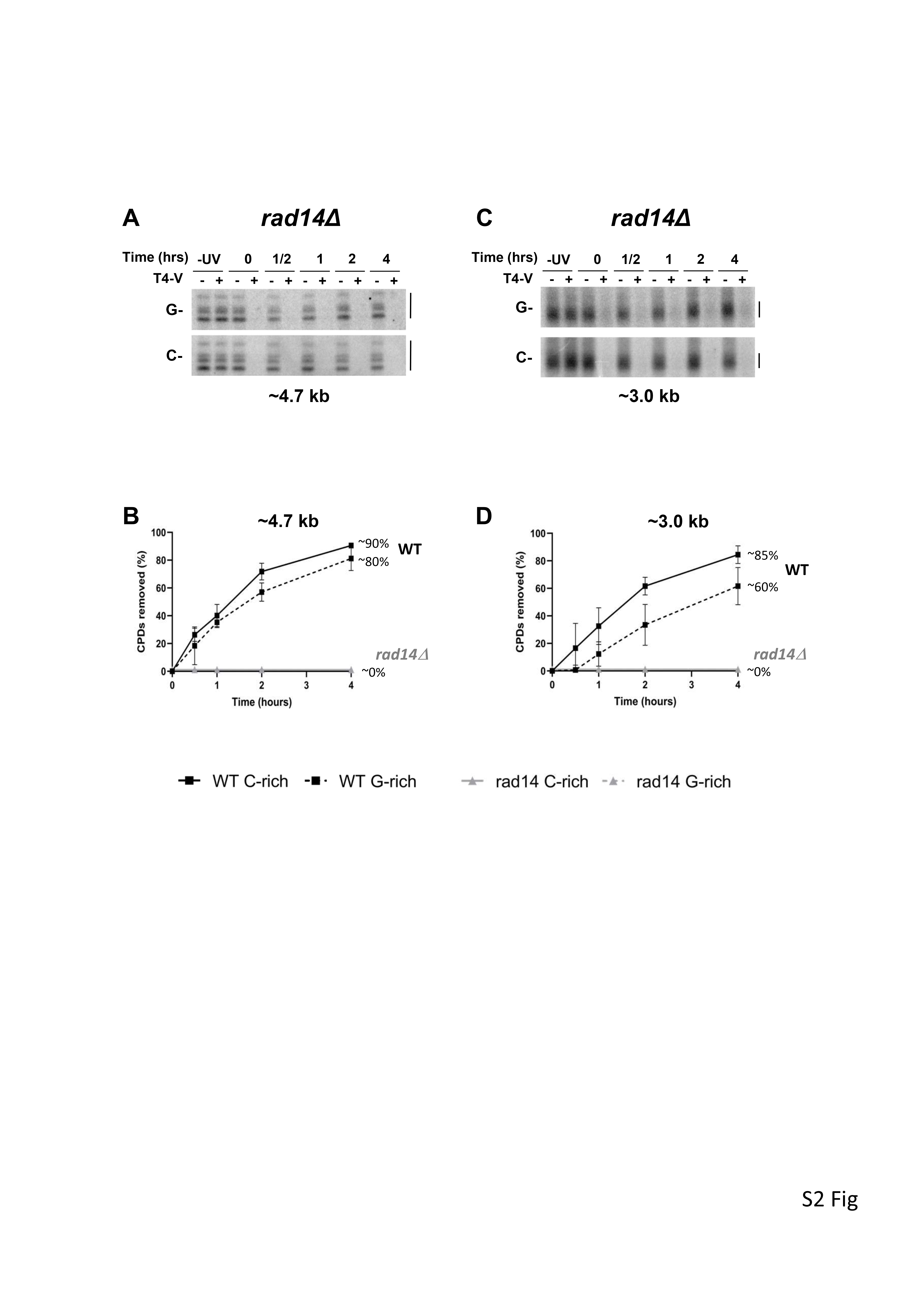

Supplement: S2 Fig — WT and rad14Δ yeast were UV irradiated, and the DNA was prepared for the T4-V assay as described in Fig 2. The band signals corresponding to the ~4.7 and ~3.0 kb fragments were measured as described in Fig 2, and the resulting means of 2 independent experiments for the rad14Δ yeast were plotted against the results obtained for the WT strain (see Fig 2). Quantifications were of CPDs for the HindIII/EcoRI group of bands (~4.7 kb), and for the bands with variable lengths (~3.0 kb) as pointed by the brackets. The results show that CPDs in the Y’-element are not repaired in the rad14Δ strain. (TIF) [file pgen.1010167.s004.tif]

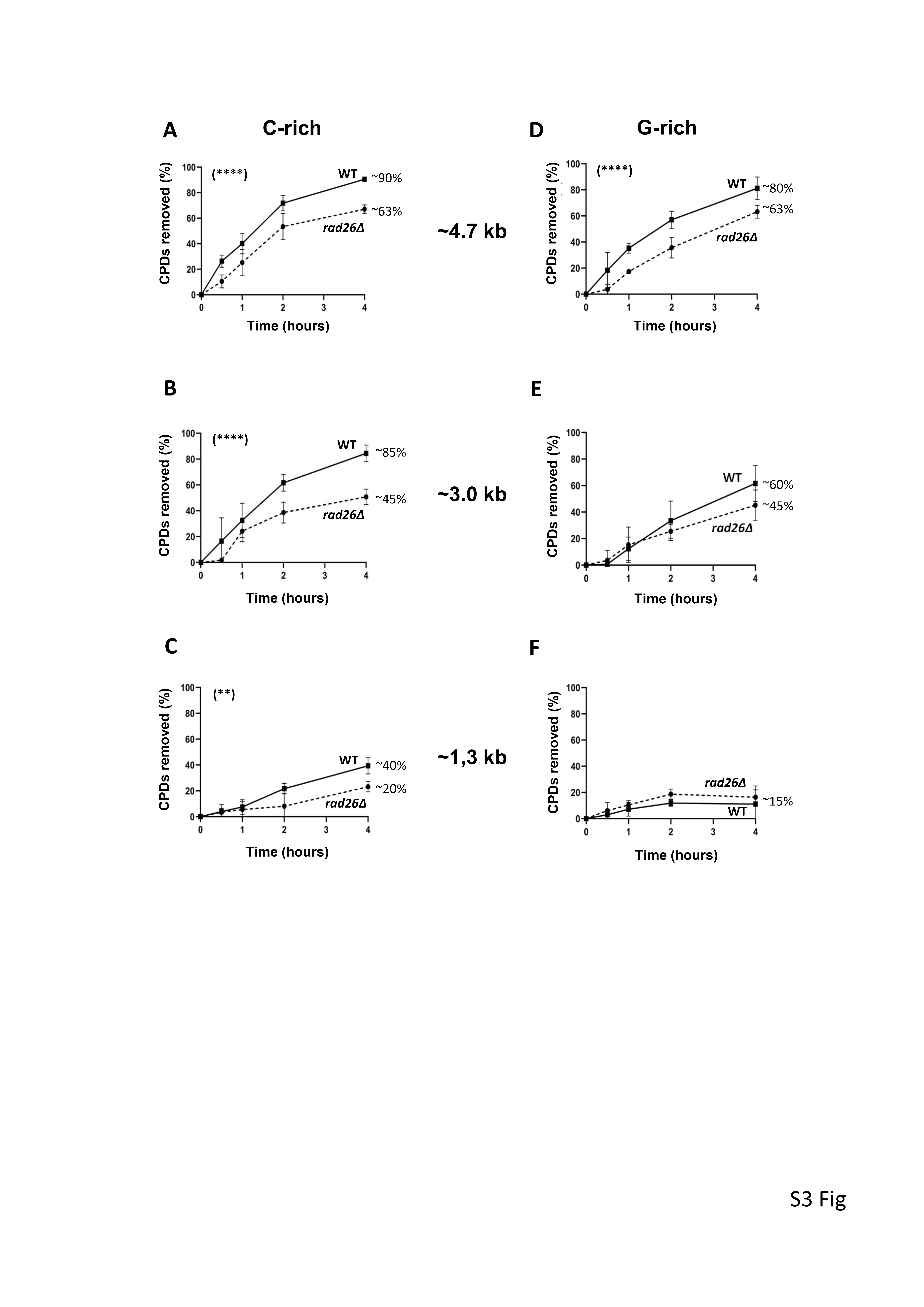

Supplement: S3 Fig — WT and rad26Δ yeast were UV irradiated, and the DNA was prepared for the T4-V assay as described in Fig 2. The band signals corresponding to the ~4.7, ~3.0 and ~1.3 kb fragments were measured as described in Fig 2, and the resulting means of 3 independent experiments ± 1SD were plotted to compare repair in the C- and G-rich strand of WT and rad126Δ yeast. P-values were calculated using a 2way ANOVA test: (**) p < 0.01, (****) p <0.0001. (TIF) [file pgen.1010167.s005.tif]

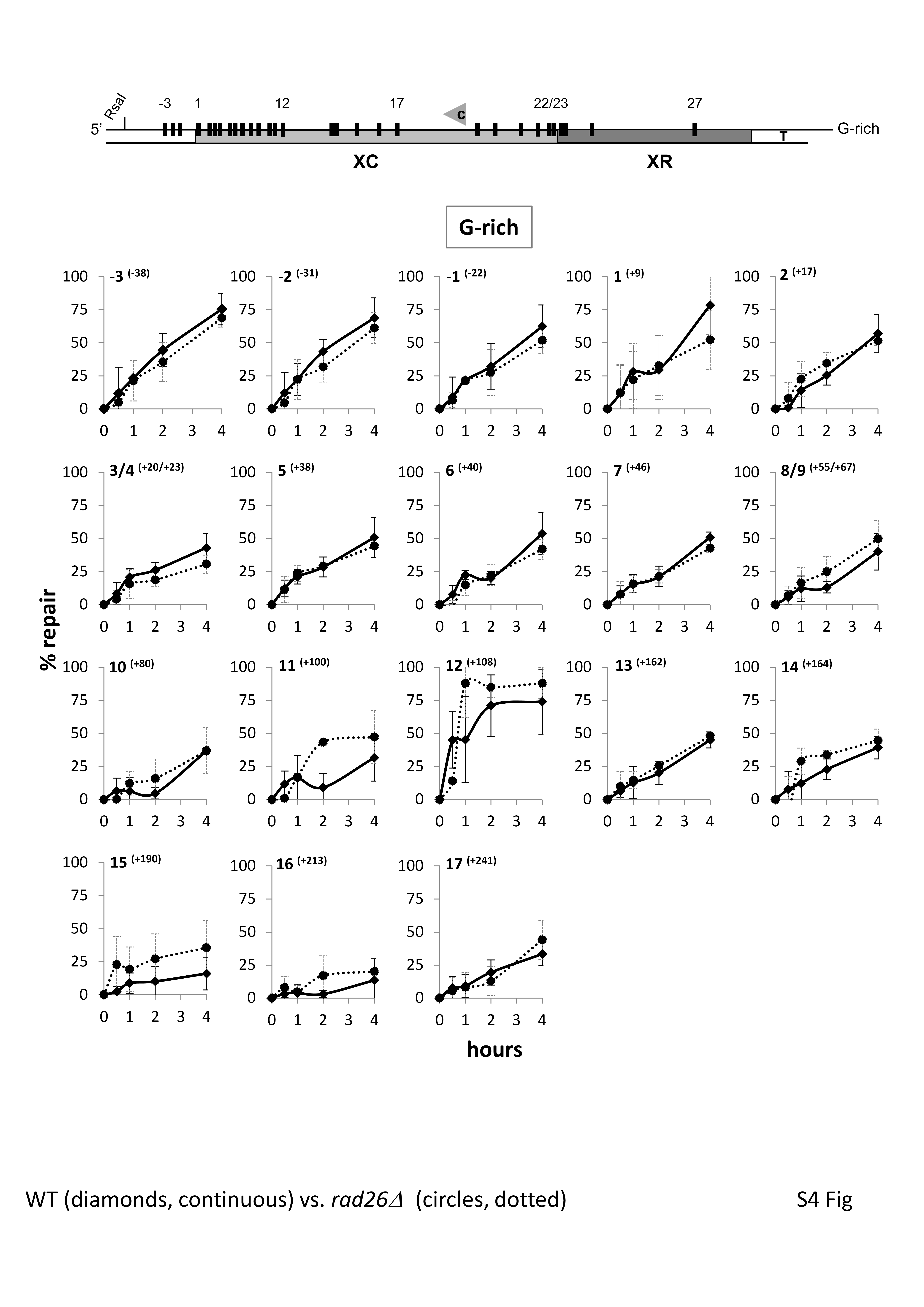

Supplement: S4 Fig — Upper panel: X-element map with the XC- and XR- regions, telomeric repeats (T) and flanking DNA (lines). Approximative DNA sequence positions of primer ‘c’, RsaI restriction site, PDs that were quantified (black bars) and numbered negatively or positively when present outside or inside of the X element, respectively. Lower panel: repair of PDs (-3 to +17) is plotted as percent of repair over time (hours); WT (diamond, continuous line) and rad26Δ (circle, dotted line). Indicated at the top of each plot is the arbitrary number of the PD, with its position on the DNA sequence in parenthesis; both numbers refer to the beginning of the X-element. Means are for 2 independent experiments, and the means ± 1SD are of 3 independent experiments. (TIF) [file pgen.1010167.s006.tif]

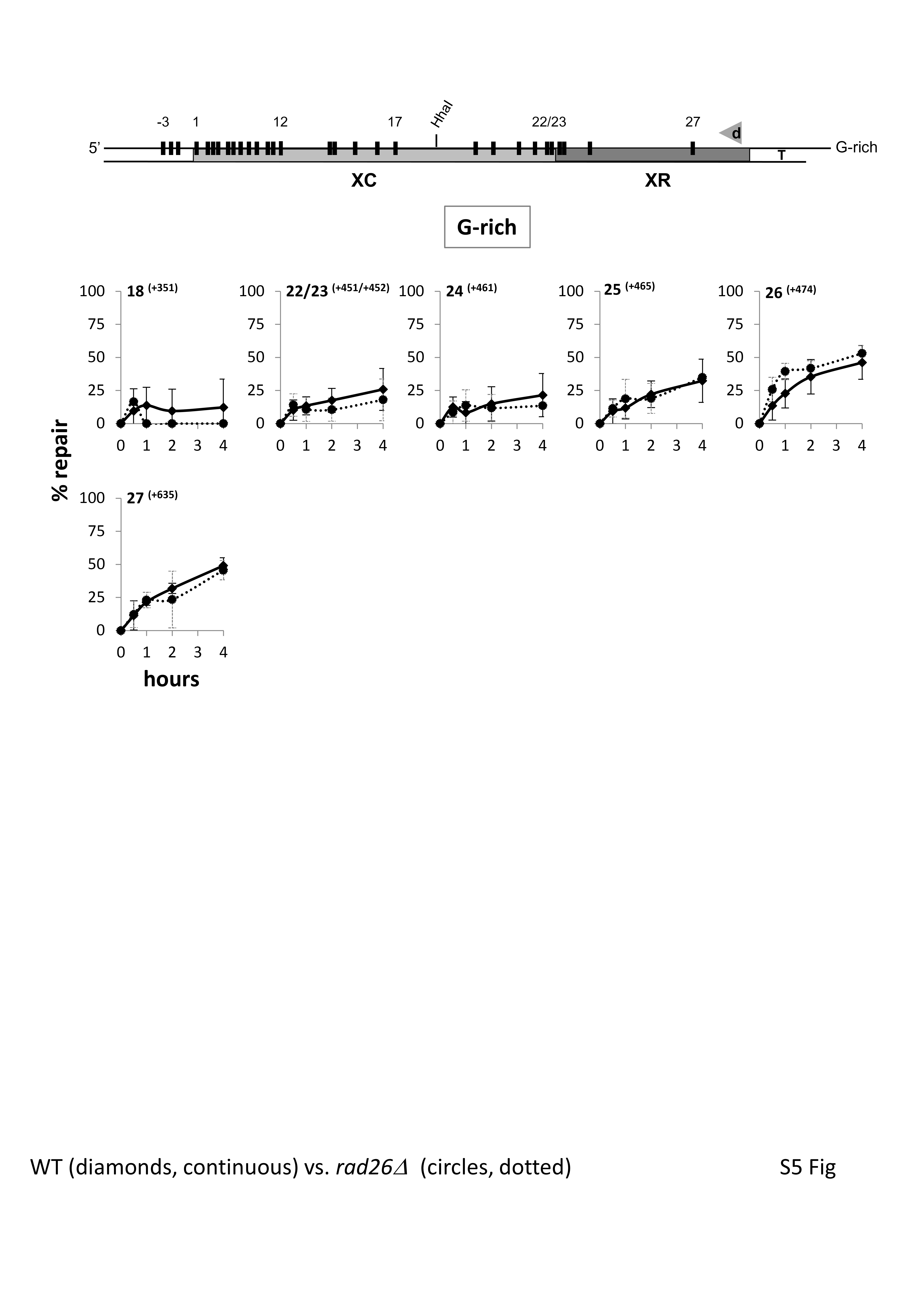

Supplement: S5 Fig — Upper and lower panels are as described in S4 Fig, with the approximative DNA sequence positions of primers ‘d’, and of the HhaI restriction site. Lower panel: repair of PDs (+18 to +27) is plotted as percent of repair over time (hours); WT (diamond, continuous line) and rad26Δ (circle, dotted line. Means are for 2 independent experiments, and the means ±1SD are of 3 independent experiments. (TIF) [file pgen.1010167.s007.tif]

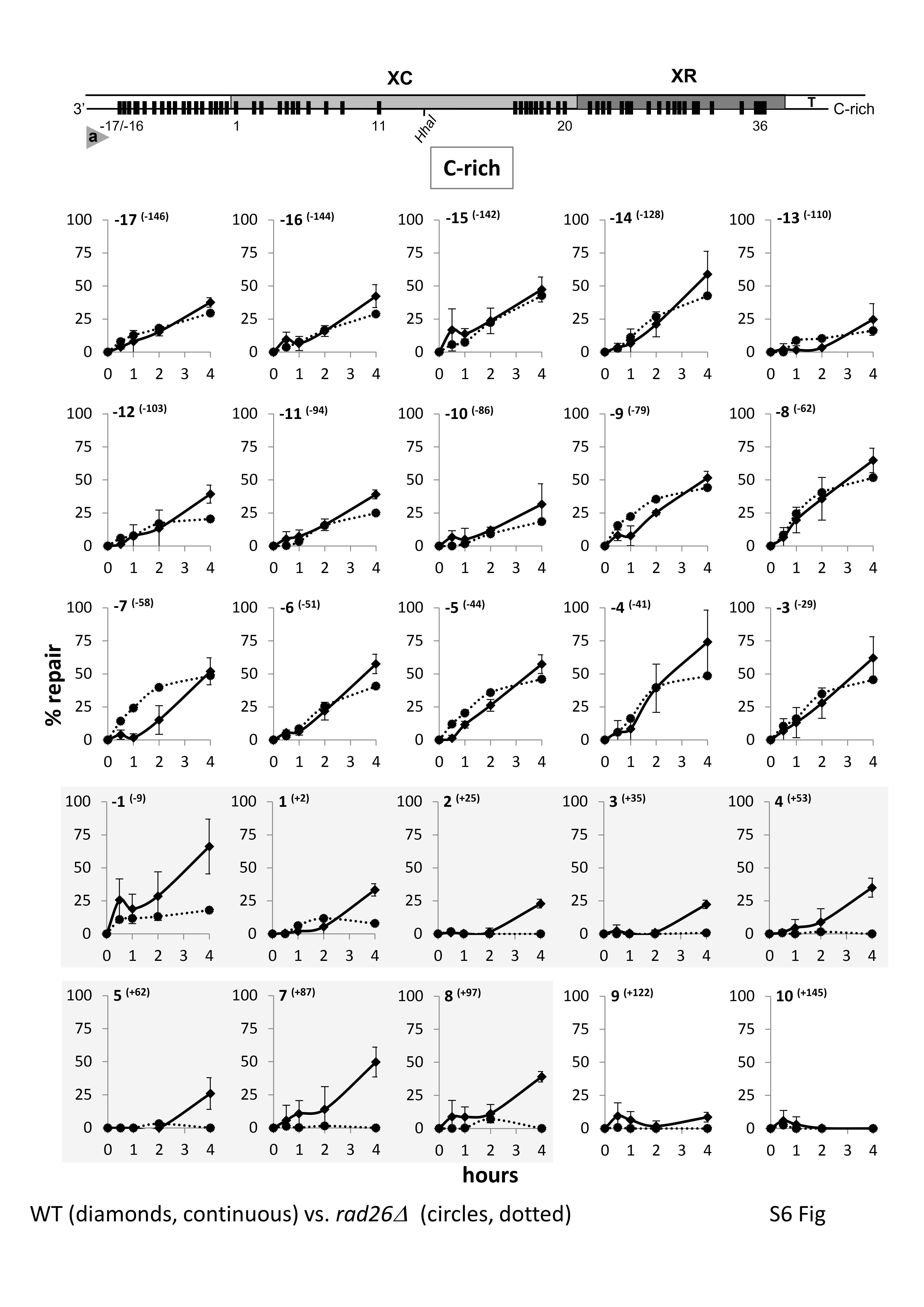

Supplement: S6 Fig — Upper and lower panels are as described in S4 Fig, with the approximative DNA sequence positions of primer ‘a’ and of the HhaI restriction site. Lower panels: PDs (-17 to +10) is plotted as percent of repair over time (hours); WT (diamond, continuous line) and rad26Δ (circle, dotted line). Means are for 2 independent experiments, and the means ±1SD are of 3 independent experiments. Grey boxes represent the region of PDs where TC-NER is observed. (TIF) [file pgen.1010167.s008.tif]

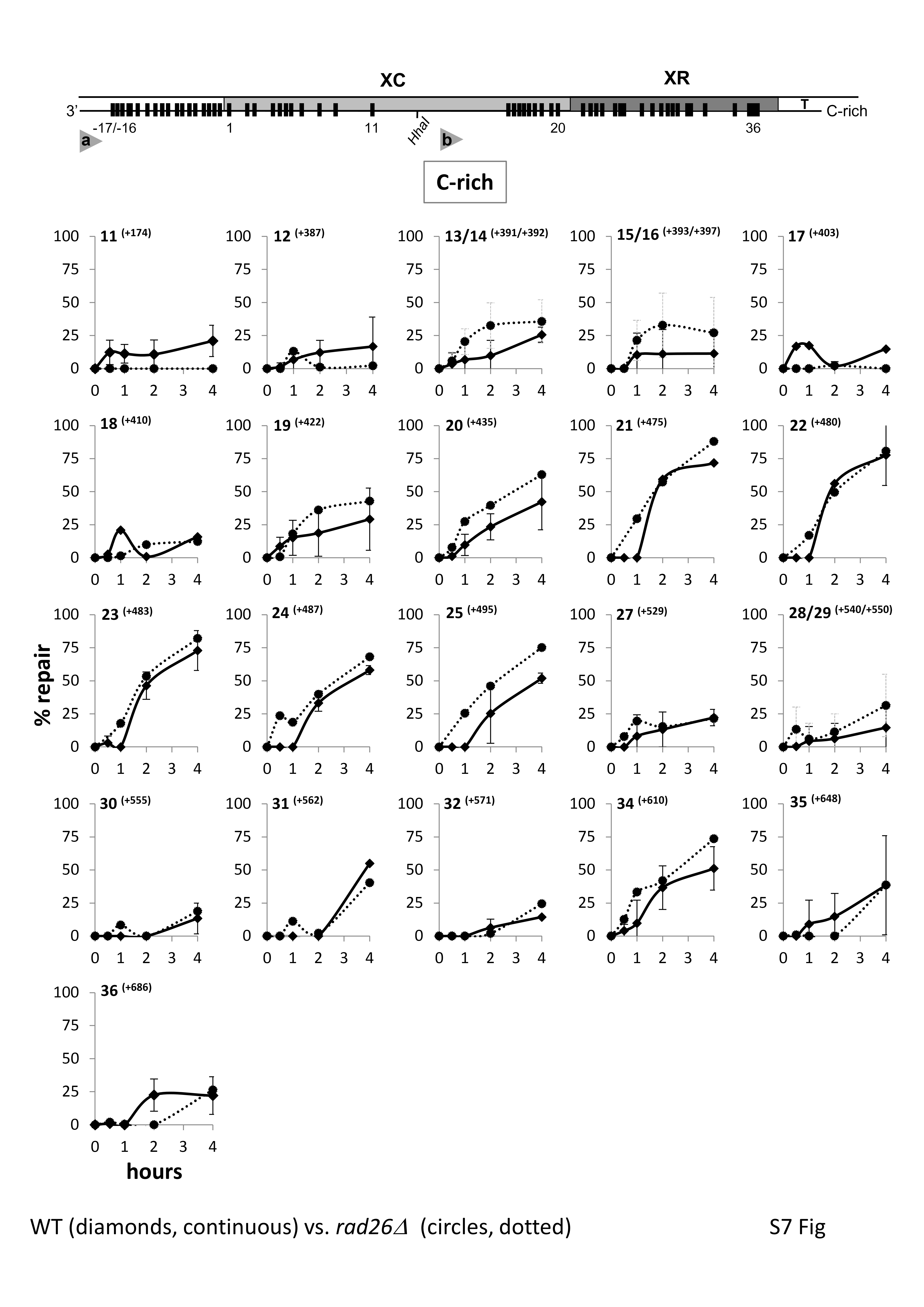

Supplement: S7 Fig — Upper and lower panels are as described in S4 Fig, with the approximative DNA sequence positions of primers ‘a’ and ‘b’, and of the HhaI restriction site. Lower panels: repair of PDs (+11 to +36) is plotted as percent of repair over time (hours); WT (diamond, continuous line) and rad26Δ (circle, dotted line). Means are for 2 independent experiments, and the means ±1SD are of 3 independent experiments. (TIF) [file pgen.1010167.s009.tif]

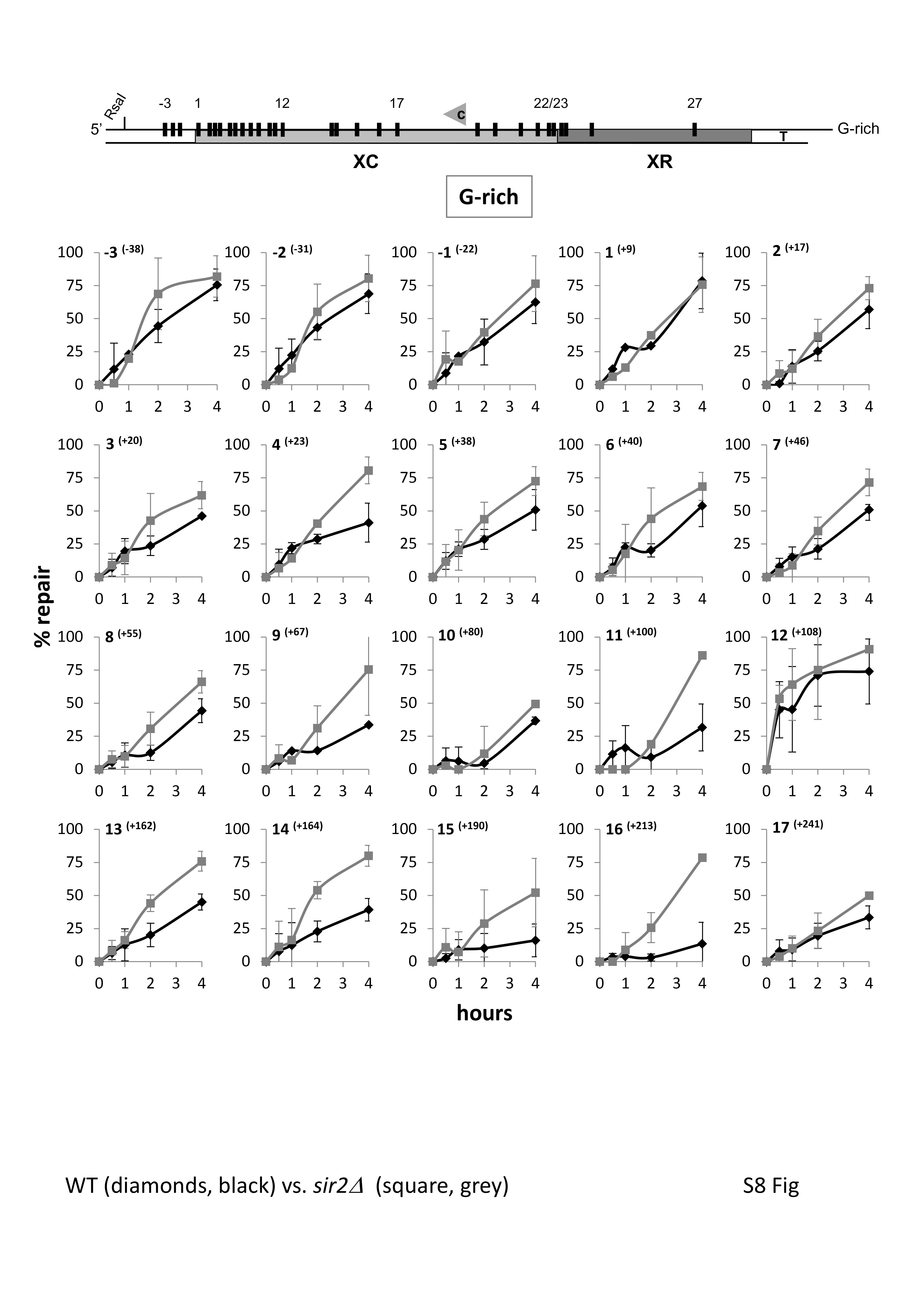

Supplement: S8 Fig — Upper and lower panels are as described in S4 Fig, with the approximative DNA sequence positions of primer ‘c’ and of the RsaI restriction site. Lower panels: Repair of PDs (-3 to +17) is plotted as percent of repair over time (hours) WT (diamond, black line) and sir2Δ (square, grey line). Means are for 2 independent experiments, and the means ±1SD are of 3 independent experiments. (TIF) [file pgen.1010167.s010.tif]

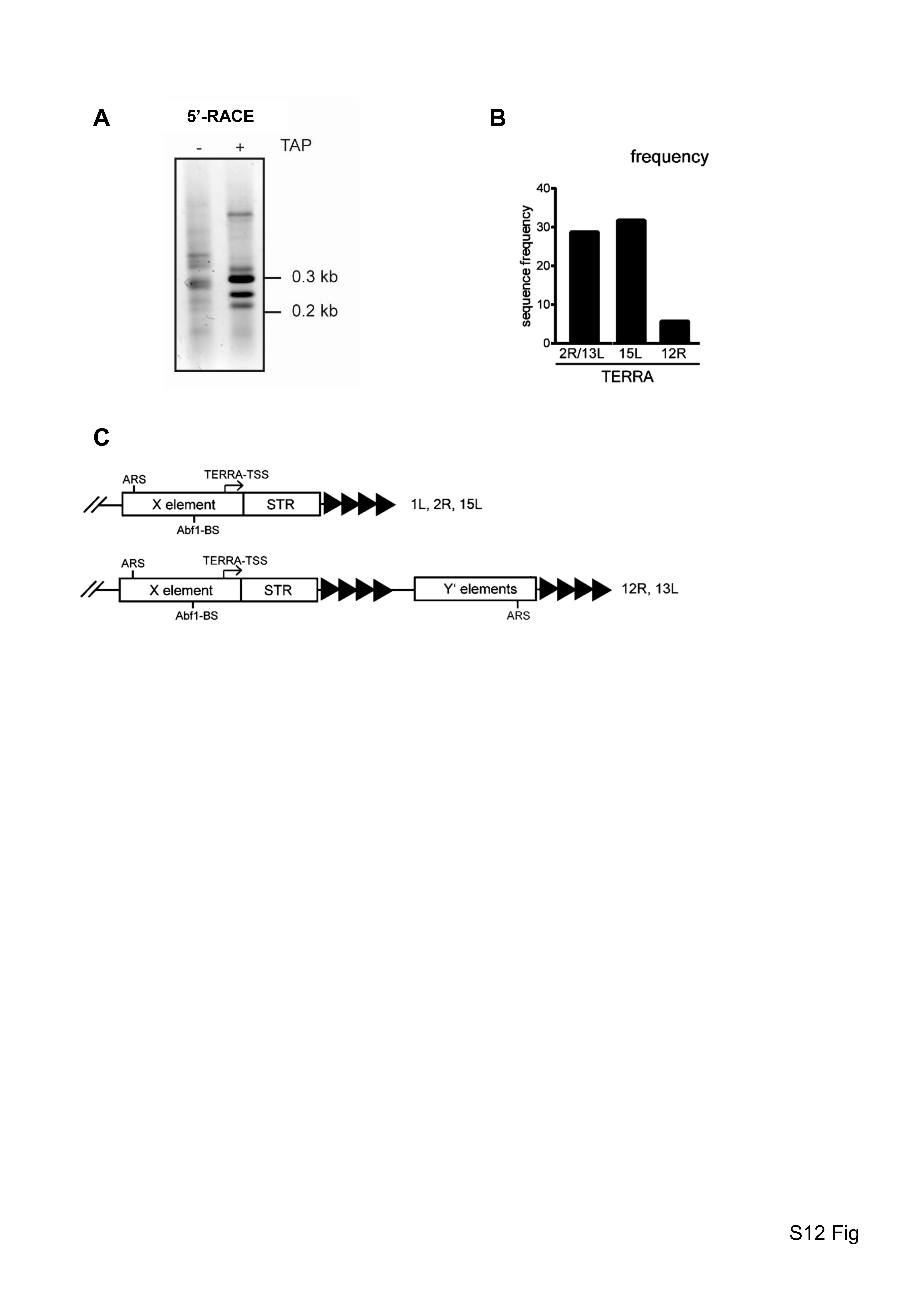

Supplement: S12 Fig — (A) 5’-RACE. The tobacco acid pyrophosphatase (TAP) was used to remove the 5’ caps of purified RNA from sir2Δ cells. A defined RNA oligo was ligated to the uncapped RNA and the product was reverse transcribed with a telomeric-repeat reverse primer. The TERRA cDNA was amplified by touch-down and nested PCR reactions, using the forward primer complementary to the RNA oligo sequence and the reverse primers specific to a subset of TERRA molecules. The PCR products were separated by agarose gel electrophoresis (+ TAP) and compared to the products of control samples (- TAP). (B) The PCR products (+TAP) were cloned into sequencing vectors. The sequences were blasted against the yeast genome and TERRA TSS were mapped (see Fig 7B). Shown are the number of hits per telomere/TERRA. (C) Schema of the sub-telomeric region with the approximative position of the TERRA TSS at the 3’-end of the X-element for telomere 1L, 2R, 13L,12R and 15L. Upper for the X only telomere and lower for the Y’ telomere. 1L TERRA TSS was previously published [42]; STR: subtelomeric repeated region; Arrow heads: telomeric repeats. (TIF) [file pgen.1010167.s014.tif]

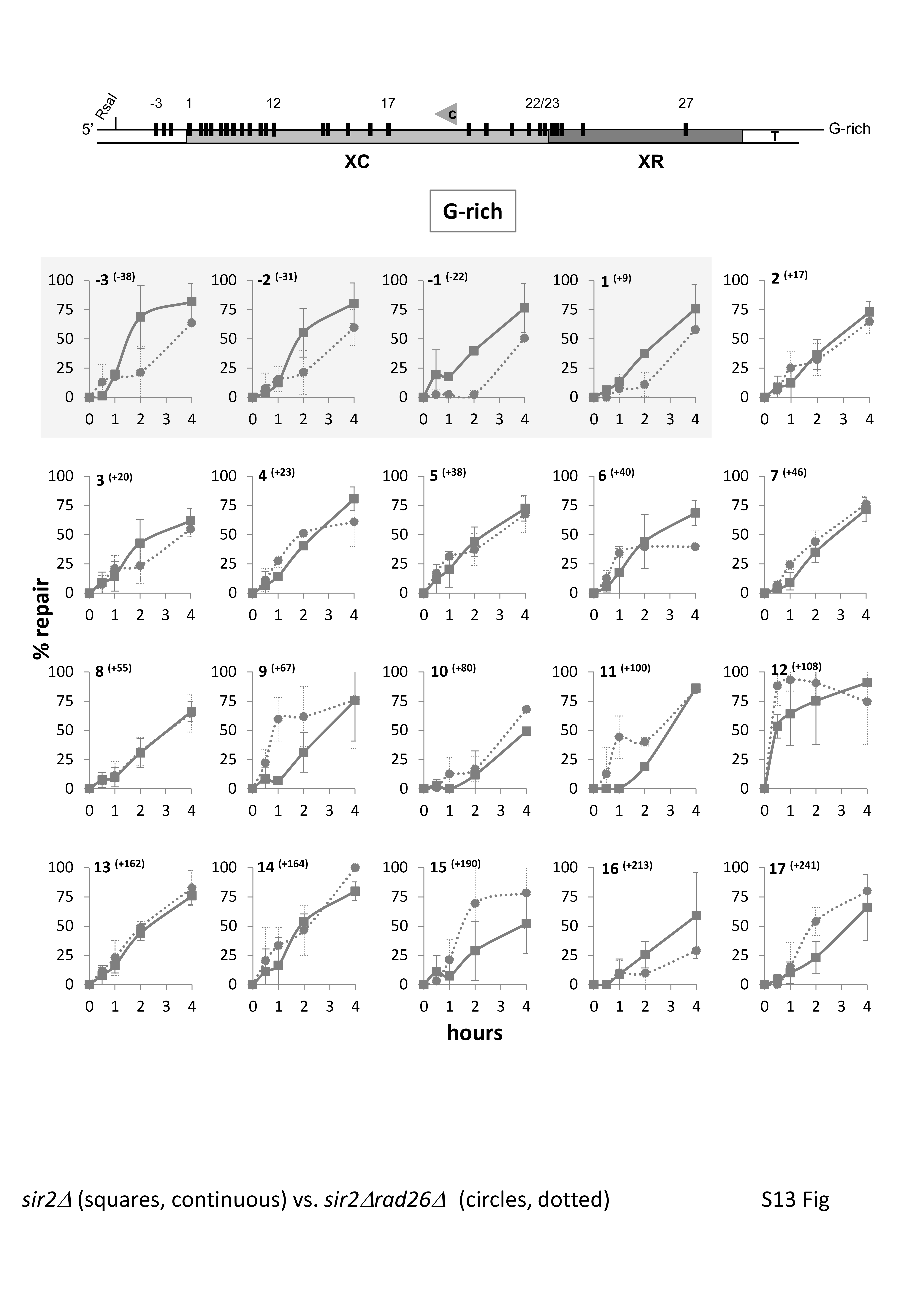

Supplement: S13 Fig — Upper and lower panels are as described in S4 Fig, with the approximative DNA sequence positions of primer ‘c’ and of the RsaI restriction site. Lower panels: Repair of PDs (-3 to +17) is plotted as percent of repair over time (hours); sir2Δ (square, continuous line) and sir2Δrad26Δ (circle, dashed line). Means are for 2 independent experiments, and the means ±1SD are of 3 independent experiments. Grey boxes represent the region of PDs where TC-NER is observed. (TIF) [file pgen.1010167.s015.tif]

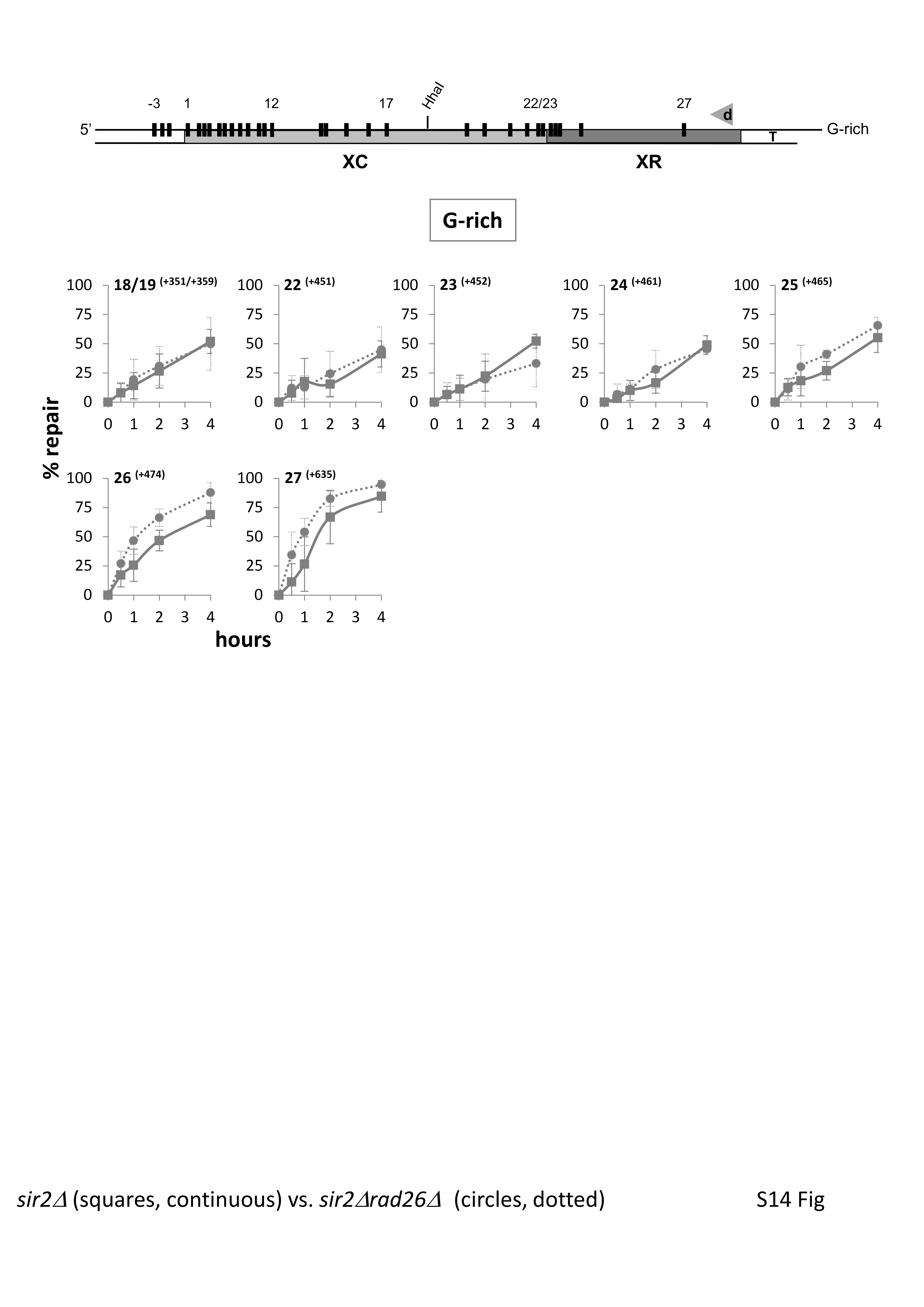

Supplement: S14 Fig — Upper and lower panels are as described in S4 Fig, with the approximative DNA sequence positions of primer ‘d’ and of the HhaI restriction site. Lower panels: Repair of PDs (+18 to +27) is plotted as percent of repair over time (hours); sir2Δ (square, continuous line) and sir2Δrad26Δ (circle, dashed line). Means are for 2 independent experiments, and the means ±1SD are of 3 independent experiments. (TIF) [file pgen.1010167.s016.tif]

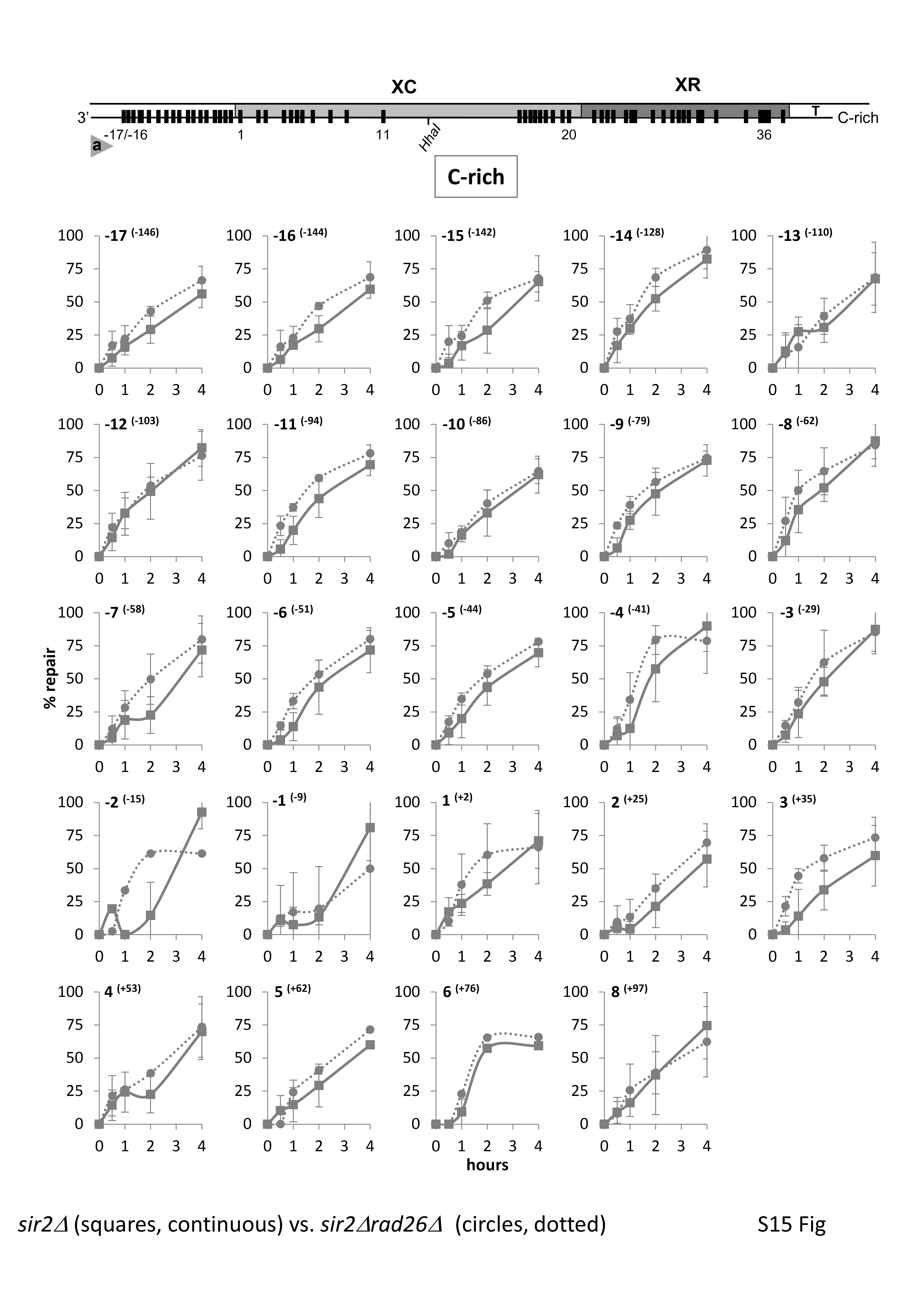

Supplement: S15 Fig — Upper and lower panels are as described in S4 Fig, with the approximative DNA sequence positions of primer ‘a’ and of the HhaI restriction site. Lower panels: Repair of PDs (-17 to +8) is plotted as percent of repair over time (hours); sir2Δ (square, continuous line) and sir2Δrad26Δ (circle, dashed line). Means are for 2 independent experiments, and the means ±1SD are of 3 independent experiments. (TIF) [file pgen.1010167.s017.tif]

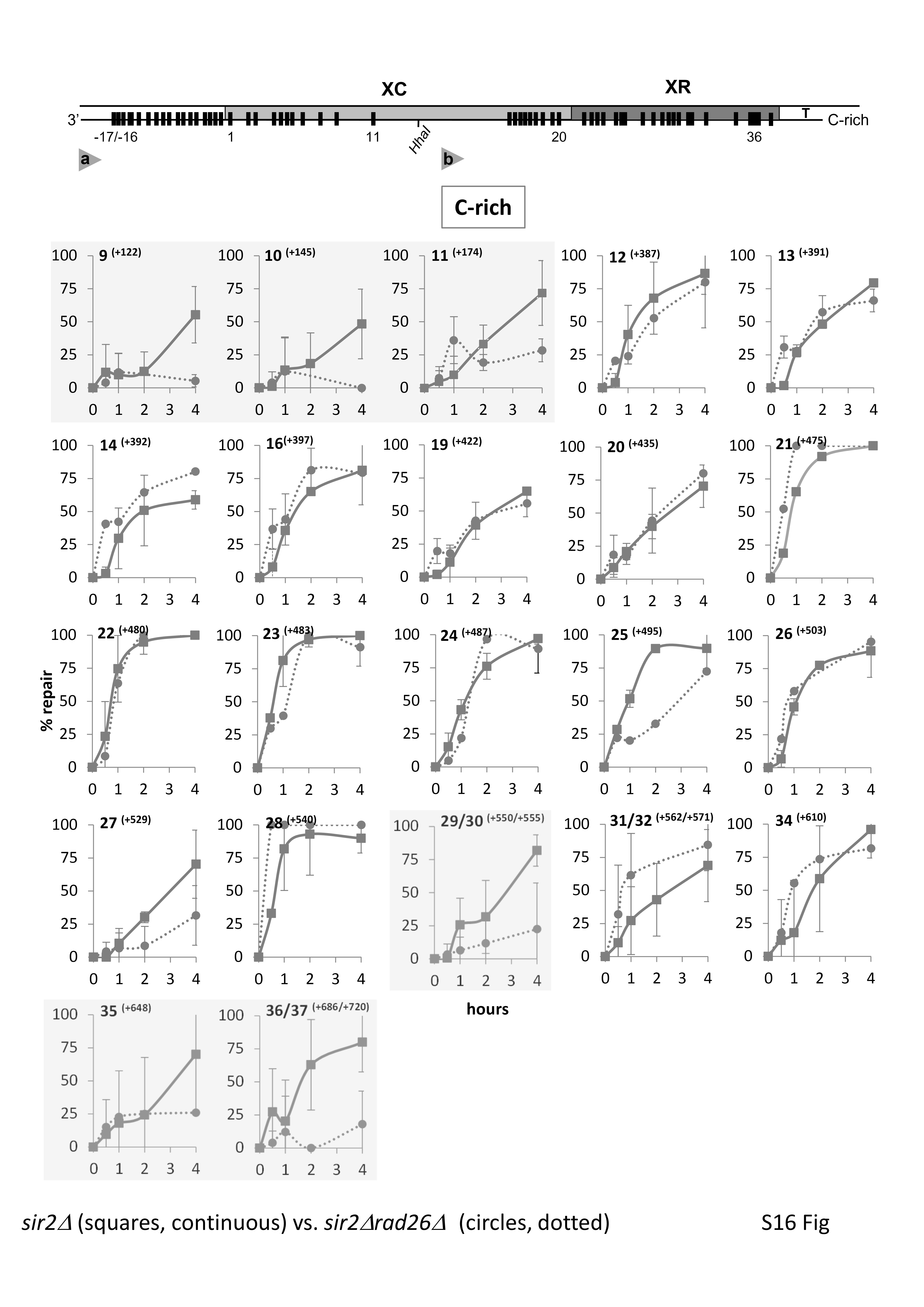

Supplement: S16 Fig — Upper and lower panels are as described in S4 Fig, with the approximative DNA sequence positions of primer ‘a’ and ‘b’ and of the HhaI restriction site. Lower panels: Repair of PDs (+9 to +37) is plotted as percent of repair over time (hours); sir2Δ (square, continuous line) and sir2Δrad26Δ (circle, dashed line). Means are for 2 independent experiments, and the means ±1SD are of 3 independent experiments. Grey boxes represent the region of PDs where TC-NER is observed. (TIF) [file pgen.1010167.s018.tif]
