# Supplementary material for: Transcription of ncRNAs promotes repair of UV induced DNA lesions in Saccharomyces cerevisiae subtelomeres
Source: PLoS Genet. 2022 Apr 29;18(4):e1010167. doi: 10.1371/journal.pgen.1010167 (PMC9106180; doi:10.1371/journal.pgen.1010167)
Supplement: Attachment — Submitted filename: Guintini Cover letter resub2 mars2022.pdf [file pgen.1010167.s020.pdf]

UNIVERSITÉ DE SHERBROOKE

Faculté de Médecine et des Sciences de la Santé
3201, rue Jean Mignault
Sherbrooke, Qc, J1E 4K8
Canada

Raymund J. Wellinger, Ph.D.
Professeur Titulaire / Professor

Canada Research Chair in Telomere Biology
Dept. of Microbiology and Infectious Diseases

Tel. 819 821 8000 ext. 75214
Fax. 819 820 6831
Email   Raymund.Wellinger@USherbrooke.ca

Sherbrooke, March 23, 2022

Editor in Chief
PLoS Genetics

Re: Re-submission of revised manuscript PGENETICS-D-21-01349R1

Dear Editor,

Concurrent with this letter, we also uploaded the necessary files for the slight correction and clarification required for the revised version of our manuscript, which is entitled: "Transcription of ncRNAs promotes repair of UV induced DNA lesions in *Saccharomyces cerevisiae* subtelomeres." Co-authored by L. Guintini, A. Paillé, M. Graf, B. Luke, A Conconi and myself (manuscript ID PGENETICS-D-21-01349R1). The submitted files concern:

- Final manuscript file
- Final manuscript file in which changes highlighted in yellow, as requested by the instructions.
- A clarified Fig. S1, including the corresponding figure legend, as requested by one reviewer.

The changes in the main manuscript file concern:

1) Rev 2 was not sure whether all the raw data were accessible to the reader.
   In response, we now added the link that leads to the Excel files in the Mendeley public repository with all the raw data (see lines 493 - 495 in the Materials and Methods section).
2) Rev 3 thought that Fig. S1 was not clear/conclusive on how the observed restriction fragment pattern was generated.
   In response, we completely edited said figure such that every observed fragment is now explicitly explained. We hope that this figure now is clear in this regard.

Importantly, none of these edits changed anything on the manuscript's message or conclusions.

We hope this version is now a final publishable version of the paper. Thank you very much for your collaboration in this matter.

Yours truly

Raymund J. Wellinger, Ph.D.
(Corresponding authors)
Professor/Professeur Titulaire
Département de Microbiologie et Infectiologie
Faculté de Médecine et des Sciences de la Santé
Université de Sherbrooke

Antonio Conconi, Ph.D.

Professor/Professeur Titulaire
