# Supplementary material for: Transcription of ncRNAs promotes repair of UV induced DNA lesions in Saccharomyces cerevisiae subtelomeres
Source: PLoS Genet. 2022 Apr 29;18(4):e1010167. doi: 10.1371/journal.pgen.1010167 (PMC9106180; doi:10.1371/journal.pgen.1010167)
Supplement: Attachment — Submitted filename: Guintini et al Ptbypt answering referees .docx [file pgen.1010167.s019.docx]

**Answers to the reviewers**

**Reviewer #1:** In this elegant study the authors use budding yeast as a model system to uncover a novel role for transcription coupled nucleotide excision…. More rapid repair on the C-rich strand, compared to the G-rich strand, in the 3.0 kb Y’ elements is consistent with a greater abundance of Sub-TERRA-XUTs compared to Sub-TERRA-CUTs…. Addition statistical analyses comparing wt and mutant strains would strengthen the conclusions. Overall, the study was very well done, and I have a few comments below.

Query 1: In the introduction it would be useful to mention that Rad26 is the yeast homolog of the human CSB gene….. and mutations in CSB cause the degenerative disorder Cockayne’s syndrome. I believe this would be of interest to the readership and underscores the relevance for human disease.
Answer: we added the following information to “Discussion” (P. 15, L. 412-416): *RAD26* is the yeast homolog of the human Cockayne’s syndrome (CSB) gene (**6**). Mutations in CSB cause the degenerative disorder, which is characterized by the lack of repair in the transcribed strand of active genes (**2-6**). Pervasive transcription of non-coding regions throughout the genome could underscore the importance of TCR and its relevance for the related disease in human.

Query 2: Figure 2. How do the authors know that they have adding saturating amounts of T4 endo nuclease; amounts sufficient to cleave all the CPDs?

Answer: This assay is widely used, and it was previously applied in several studies. The requested control experiments were done when the assay was introduced several years ago. The T4 endo V assay is commercially available and, like other research groups that study the formation and repair of CPDs using T4 endonuclease V, we follow the established manufacturer’s recommendations. This note was added in “Material and methods”: P. 17, L.467-468.

Query 3: The authors note that the formation of photoproducts depends on sequence. Are the sequences in the Y’ and X elements known?

Answer: Yes, both sequences have adjacent pyrimidines that are potential sites for the formation of CPDs. The sequences can be found on the <https://www.yeastgenome.org>.

Query 4: The graphs in Figs 2-4 lack statistical comparisons between WT and rad26 null strains. Statistical comparison between C-rich and G-rich strand repair would also strengthen the conclusions, particularly for Figs 2B and 2C.

Answer: We added the statistical values to the repair measurements for the Y’-element: a) C-rich vs. G-rich strand, p-values are in **Fig. 2** and in the corresponding figure legend: P. 26, L. 732-733; b) WT vs. *rad26Δ*, p-values are in the new **Fig. S3** and in the corresponding figure legend: P. 31, L. 864-869. We added the statistical values to the repair measurements for the X-element, WT vs. *rad26Δ*: a) Legend of **Fig. 3C**: P.27, L. 749-751; b) **Fig. 4C** and corresponding figure legend: P. 27, L. 765-767; c) **Figs. 5C** and **5D**, and corresponding figure legends: P. 28, L. 780-783; d) **Figs. 7C** and **7D**, and the corresponding figure legends: P. 29, L. 814-817.

Query 5: Do the authors have an estimate of how many CPDs were induced on the various elements and fragments?
Answer: The number of CPDs were added for each fragment of the Y’-element, and the information is in the legend of **Fig. 2.** P. 26, L. 723-730. For the X-element the numbers of PDs are described in **Figs. S4, S5, S6, S7, S9, S10**.

Query 6: Fig 2. Are both strands transcribed to a similar degree? Are Sub-TERRA-XUTs similar in abundance to Sub-TERRA-CUTs? The authors reference a manuscript that indicate the former is more abundant.

Answer: The C-rich strand is more transcribed than the G-rich strand; ref. 46; P. 9, L.229-231; P. 13, L. 355-356.

Query 7: Fig 5. Statistical comparison between repair in WT and sir2 null strains would strengthen the conclusions.

Answer: We agree with the Referee. The statistics were added as requested. Please see answer to Query 4 for details.

Query 8: Treatment with general transcription inhibitors (i.e. thiolutin) are typically used as controls in TCR assay. Have the authors tried transcription inhibitors?

Answer: Sometimes α-amanitin and thiolutin were employed to characterize transcription-dependent-nucleotide excision repair. Nonetheless, it is also reported that these drugs could be unspecific inhibitors of transcription, and that thiolutin could have toxic effects on the physiology of yeast cells (Trcek, Cell 2011; Pelechano, Yeast 2008). Therefore, while the proposed experiments are feasible, we believe that they may not add more conclusive evidence for TCR.

Query 9: Discussion. It is not clear why Sir2 would impair NER. Do the authors have a suggestion? Is there any evidence that Rap1, Rif1 and Sir proteins impair lesion recognition by the Rad4-Rad23 complex in GGR?

Answer: Sir proteins are present in the *HMR* locus and in the X-element, but they are absent in the Y’-element. In *sirΔ* yeast NER efficiency is only affected (increased) in the *HMR* locus and in the X-element. Our previous results (Guintini et al., NAR 2017) suggest that the stabilization of nucleosomes by Sir3 inhibited NER. In fact, PDs could be exposed to NER during transient shifts in nucleosome positioning (Wellinger, EMBO 1997). Slow repair in the X-element heterochromatin could result from the reduced mobility of nucleosomes. Part of this comment was added to “Discussion”; P. 14, L. 377-381.

Query 10: Discussion. While the manuscript is focused on NER in yeast, it would be interesting to compare results with what is known regarding NER at telomeres in human cell lines, especially since genetic defects in TCR and GGR genes give rise to genetic disorders (XP, Cockayne’s syndrome).

Answer: In human cells, the estimate of NER efficiency at telomeres remains controversial. An early study indicated that cells efficiently removed PDs from telomeric DNA (Kruk, PNAS 1995). This conclusion was supported by findings obtained with fibroblasts expressing exogenous telomerase, which showed that CPDs in telomeres were repaired faster than CPDs in the bulk of the genome (Parikh Nature Com. 2015). In contrast, Rochette et al. found that CPDs are not repaired in telomeres and that cells tolerate persistent high levels of damaged telomeric DNA. Our results indicated that PDs in yeast telomeres are repaired mostly by GGR, albeit at slow rate. As requested, this information was added to “Discussion”; P.15, L. 405-412.

**Reviewer #2:**

Query 1: … (Guintini et al., NAR, 2017), the authors showed that Rad26 contributed to repair of UV damage at Y’ elements at yeast telomeres. Since this finding overlaps to some degree with the current report, it would be important for the authors to distinguish the major advances of the new study, relative to these previous findings.

Answer: In Guintini et al. (2017), we focused on the uncharacterized, or dubious, ORFs that are present on both DNA strands of the Y’ element. These ORFs are more abundant in the telomere distal DNA fragment (4.7 kb fragment). To test the possibility that the change in NER efficiency was related to transcription of the uncharacterized ORFs, we followed repair in the *rad26Δ* strain, without separating the C- and G- rich DNA strand. The results (Guintini et al., 2017; Supp. Fig. 4) showed that about 17% and 13% repair of CPDs was Rad26 dependent, for the 4.7 and 3.0 kb fragments, respectively. These results prompted us to separately investigate TCR in the C- and G- rich DNA strand, to determine if there was a correlation between TERRA transcription and repair efficiency of CPDs (**Figs. 2** and **Fig S3**). As requested by the reviewer and to clarify this point, the above comments were added to “Results”, P. 8, L. 203-208.

Query 2: … Since some of the differences in repair attributed to TC-NER are relatively modest, it might be helpful to try some of these experiments in a rad16 mutant background.

Answer: We agree with the Referee that in a number of sub-telomeric domains there is only a modest participation of Rad26 dependent repair of PDs. Consistently, considering the heterochromatin-like structure of these regions, it was predictable that PDs were mostly removed by GGR. Moreover, TCR correlates with transcription activity; namely, high transcription rates are associated with high repair rates (Hu et al. 2015, *Genome-wide analysis of human global and transcription-coupled excision repair of UV damage at single-nucleotide resolution*). TERRA transcription levels are extremely low in both, the X-element where it is repressed by Sir2/3/4, and in the Y’-element where it is repressed by the Rap1 recruited Rif1/2 complex (Luke, EMBO Rep 2011). Consequently, in the two sub-telomeric regions there is only a modest participation of Rad26 dependent NER. We thank the Referee for the comment, which prompted us to clarify our results. The above comment was added to ”Discussion”: P. 16, L. 432-437. However, we did not initiate the whole sets of experiments with *rad16Δ* and rad16/26Δ mutants. Although it is a valid suggestion, completion of these experiments would require several months of work, the results of which would only add minor information to our controls.

Query 3: The high-resolution repair analysis of the Tel-15L telomere (e.g., figures 3, 4,etc) is beautiful ….the interpretation of the data is limited to some degree by the lack of accurate ncRNA/transcription data for this genomic region. It might be helpful to try to generate this data to assist in the analysis.

Answer: In response to the Referee’s suggestion, we teamed up with the laboratory of B. Luke that generated the map of transcription start sites for ncRNAs in a number of X-telomeres (see new Fig. 6B and Fig. S8). The results show that the TERRA transcription start-sites are found in the same region of the X-element of different telomeres. This set of data quite clearly supports the connection between TERRA transcription and the repair of PDs at the end of the X-element.

Query 4: … do a similar high-resolution analysis of the Y’ element, in order to see whether the TERRA transcript corresponds with the regions of high TC-NER in these sub-telomeric regions.

Answer: As illustrated in Figure 1A, Y’-elements are transcribed on both DNA strands, where transcription is randomly distributed, although more predominant on the C-rich strand. The results of the T4-endo V assay (**Fig. 2**) show a nice correlation between density of transcription and Rad26 dependent repair of CPDs. We wish to emphasise that the highly conserved DNA sequences of all Y’ elements (about 25 in the genome of which approximately 16 are terminal Y’s) do not allow to design probes that are specific for one chromosome end. Consequently, high-resolution analysis for a single, specific Y’ element cannot be done. Both, the random distribution of lncRNA transcription on both DNA strands, and the impossibility to design primers for the high-resolution repair-mapping of PDs for a single chromosome, all this limits the type of assay that can be applied to study repair in native telomeres.

Query 5: The impact of the study could be improved by seeing if the TC-NER activity detected in yeast is also associated with non-coding TERRA expression in human telomeres. This could be analyzed Bioinformatically using published XR-seek data from XPC mutant cells (e.g., Hu et al., 2015).

Answer: We performed bioinformatics analyses of the human-related data generated by the XR-seq genome wide mapping. Available data were extracted from the Gene Expression Omnibus database (GEO) using the accession number GSE67941 (Hu et al., 2015). We used the CPD and (6-4)PP mapping data for the NHF1 wild-type cells, the CSB and the XPC mutant cell lines. We visualized the distribution of the aligned reads for the two strands of telomeres of all human chromosomes (hg19), using the UCSC genome bro. We did not find reads for telomere sequences: e.g., Tel 7L: 0-10000.

Nonetheless, we added a comment of NER in the telomeres of human cells in “Discussion”: P. 15, L. 405-412.

**Reviewer #3:**

Major points:

Query 1: The authors talk exclusively about NER, …. The authors could use an NER mutant in their system to show that all repair is proceeding via NER.

Answer: We acknowledge that in the submitted manuscript, the interpretation of our data lacked information on the repair pathways that could participate to remove UV induced DNA lesions from the subtelomeres. In the new, revised version of the manuscript we indicate that Rad14 is a specific and essential NER protein-factor, which participates in the recognition step of UV induced DNA damage. Therefore, r*ad14Δ* is the NER mutant that we used as control; see P. 8, L. 196-198. We added a supplementary figure (new **Fig S2**) showing that all CPDs in both DNA strands of the Y’-element are not repaired in *rad14Δ* yeast. We hope that this new figure meets the Reviewer’s request.

Query 2: …. **A**) how deletion of SIR2 actually improves repair, …. **B**) which repair pathways are involved, and **C**) whether increased transcription is responsible. **D**) Are *sir2∆* cells more resistant to UV?

Answer: **A)** Sir proteins are located in the *HMR* locus and in the X-element, but not in the Y’-element. In *sirΔ* yeast, NER efficiency was only affected (increased) in the *HMR* locus and in the X-element (Guintini et al., NAR 2017). Moreover, we indicated that the stabilization of nucleosomes by Sir3 could have inhibited NER. This conclusion was based on a different study which showed that PDs could be exposed to NER during transient shift of nucleosome positioning (Wellinger and Thoma, EMBO 1997). The slow repair of UV photoproducts in the X-element heterochromatin could result from Sir proteins-dependent reduced mobility of nucleosomes. A similar comment has been added to “Discussion”: P. 14, L. 377-381. **B)** As summarized in the **new Fig. 6A**: in *sir2Δ* (light grey columns), GGR efficiency clearly increased in clusters II, IV, V, VII and VIII (G-rich strand), and in clusters -I, -II, -VII, -VIII, -IX, -X (C-rich strand), where there was no TCR; in WT (*SIR2^+^*) (dark grey columns), PDs in clusters -III, -IV and -V (C-rich strand) were mostly removed by TCR (compare dark grey columns with black arrows), implying that these regions were transcribed. However, in *sir2Δ* yeast the same clusters of PDs were removed by GGR, because there was no TCR (repair of PDs was similar in *sir2Δ* and *sir2Δrad26Δ* strains), implying that transcription ceased. Hence in *sir2Δ*, GGR efficiency also increased in clusters -III, -IV and -V (C-rich strand); **C)** In *sir2Δ*, transcription was induced (or increased) around clusters I (G-rich) and -VI, -XIa and -XIIa (C-rich), which triggered TCR (white arrows). At the same time GGR efficiency also increased in cluster -XIIa, but only slightly increased in clusters -VI and -XIa. In summary, in WT (*SIR2^+^*) most clusters of PDs were removed by GGR, except for cluster -III, -IV and -V that were removed by TCR. In *sir2Δ* the efficiency of GGR increased in most of the clusters, together with transcription triggered TCR in clusters I, -VI, -XIa and -XIIa. The results also show that there could be heterochromatin gaps lacking Sir2, like the regions surrounding clusters I/-III, III/-V, and VI, where the efficiency of GGR was similar in WT and *sir2Δ* strains. **D)** Previously, we showed that *sir1, 2, 3 and 4Δ* cells are more resistant to UV irradiation (Toussaint et al. Mut Res 698, 2010; Figs 1A and 1C).

Other points:

Query 1: Line 169: It is unclear how 4.4, 4.7, and 5.0 kb bands are obtained. Perhaps a diagram could help.

Answer: we added a restriction map of the Y’-elements in a supplementary figure (new **Fig S1**).

Query 2: It is mentioned that NER efficiency decreases in a centromere-to-telomere direction … It is difficult to compare the 4.7, 3.0, and 1.3 kb fragments because the amount of initial damage decreases with decreasing fragment size. Also, as mentioned by the authors, NER efficiency depends on both DNA sequence and length, and these two variables are not controlled for.

Answer: The formation of DNA damage depends on the DNA sequence; PDs form preferentially in TT >TC > CT > CC (Ravanat et al., Photochem. Photobiol 2001). Nonetheless, over several hundred of nucleotides the presence and type of adjacent pyrimidines becomes randomly distributed (except for pyrin and pyrimidine rich regions). The number of PDs that forms in irradiated DNA is indeed DNA length dependent, but so is the number of NER complexes. Taken all these considerations together, the efficiency of NER has been previously compared between DNA fragments of different lengths (e.g., Conconi et al., PNAS 2002; Fig 4): P. 9, L. 235-237. As implied by the Reviewer it is important to specify that the number of CPDs per kb was very similar in all 3 Y’ DNA fragments (C-rich strain): for the 4.7, 3.0, and 1.3 kb, they were 0.67±0.07, 0.69±0.38 and 0.58±0.08, respectively (This is now described in Fig. 2).

Query 3: In Fig. 2, why are there different banding patterns in 2A and 2D, and also 2B and 2E? It is unclear why deletion of RAD26 would cause such an effect.
Answer: We thank the Referee for this remark. However, deletion of RAD26 does not cause changes in fragment lengths. Different migration (voltage and time) and agarose concentrations, between experiments, resulted in slight differences in the separation of restriction fragments. These changes in gel electrophoresis conditions, between the experiments, were made to optimize the banding pattern for the T4-endo V assay.

Query 4: In Fig. 3, why are natural pause sites (*) on the G-rich strand different between WT and rad26∆? Similarly, comparing Fig. 5 with Fig. 4, why are the natural pause sites on the C-rich strand different for sir2∆ than WT?
Answer: We do not know why 1 out of 3 Taq-polymerase natural pause sites in the G-rich strand is not present in *rad26Δ*, and yet is present in *sir2Δ* and *sir2Δrad26Δ* strains. Also, we cannot explain the slight differences in natural pause sites between the C-rich strand of *sir2Δ* and WT. Thus, the importance of the “-UV lane”, which allows to subtract the parasite bands from the pattern of bands resulting from bona fide DNA polymerase arrests at pyrimidine dimers.

Query 5: In Fig. S1B (Fig. S4B of the re-submitted version), where are PDs 19-21? Where is cluster VI for Figure (former) 6A/B?

Answer: For *rad26Δ* and *sir2Δ* (G-rich DNA strand), and for cluster VI (*sir2Δrad26Δ*; PDs 19-20), in all 3 replicates the signals were too weak for quantification.

Query 6: Fig. S1A/B (Fig. S4A and S4B of the re-submitted version) maps of PDs do not match that of the map in Fig. 3B (note the position of the PDs on either side of the XR/XC boundary).
Answer: We thank the Referee, and we made the requested corrections to the figures. We note that all PDs were added to the maps of Supplementary Figures, whereas in the Figures of the main text PDs 19-21 were not added (see answer to query 5).

Query 7: From the gel shown in Fig. 4A, it appears that repair of PDs -17, -16, and -15 is defective in rad26∆, but the corresponding graphs in Fig. 4 and (former) Fig. S2 say otherwise. Can the authors explain this?
Answer: In Fig. 4A it appears that PDs -17 to -15 are repaired faster in WT than in *rad26Δ*. This results from a flaw in gel migration of lane 4 hours (see white streak). When we quantified the data, we avoided the white streak. Please note that Figs. 4C and S5 (of the resubmitted manuscript) represent the average of independent experiments.

Query 8: Typo in legends for Fig. 5/6: “The DNA was prepared and ASSED…”

Answer: We thank the referee and made the corrections.

Query 9: Since the clusters are defined differently for WT (Fig. 3 and 4) than sir2∆ (Fig. 5) (i.e. they do not correspond to the same the regions of DNA), how were the WT and sir2∆ clusters compared in Fig. 5C and 5D?
Answer: We thank the referee for the remark, and we added the following information to “Results”: For both strains, the clusters of PDs were defined from the repair curves described in **Figures S6** and **S7** (G-strand: I to VIII, C-rich: -I to -XII): P. 11, L. 276-278.

Query 10: Fig. 7 is discussed before Fig. 6.

Answer: We changed the order of the two figures.

Query 11: In the Fig. 6 legend (Fig. 7 of the re-submitted version), it should be sir2∆ (square, continuous line) and sir2∆ rad26∆ (circle, dotted line). For all graphs in all figures, there should be legends defining the lines in the figures themselves (not just a description in the figure legends).

Answer: We thank the referee; changes were made as requested.

Query 12: Typo on line 302: “extend” should be “extent”.
Answer: We thank the referee. Changes were made.

Query 13: On lines 307-308, the authors propose that increased TERRA transcription increases TCR of cluster -XIa and cluster -XIIa. If this is indeed the case, and the effect is caused by a single transcript (i.e. TERRA), why would the intervening cluster -XIIb not also be affected?
Answer: Multiple transcription start sites (TSS) were found in mice, but one distinct TERRA TSS is proposed for yeast sub-telomeres (see Fig. 6B). We hypothesize that the discontinuing participation of TCR (clusters - XIa and - XIIa) could result from the local binding of proteins that, with the Sir complex, form the telomeres. In the absence of Sir2, TERRA transcription promotes TCR, but GGR is still required to remove DNA damage in the presence of telomere proteins: P. 12, L. 323-330.

Query 14: It is unclear whether Tel1L TERRA transcription can be directly compared with Tel15L. Could the authors map the transcriptional start site for Tel15L using the same approach as Pfeiffer and Lingner?
Answer: We teamed up with the laboratory of B. Luke who performed the experiments requested by the Referee (see new **Figs. S8** and **6B**). The results show that the TERRA transcription start-sites in the X-element of different telomeres fall into a very narrow region of the X-element, as we anticipated before.

Query 15: In Fig. 7, it is unclear what the black and white arrows mean.

Answer: The two arrows indicate the percent of pyrimidine dimers that were repaired after 4 hours by the TCR sub-pathway; white for WT (*SIR2^+^*) and black for *sir2Δ* cells: legend of new Figure 6.

Query 16: Recent work from d’Adda di Fagagna’s lab suggests that telomeric damage-induced long non-coding RNA (tdilncRNA) is important to repair damage at telomeres. Can the authors speculate whether such RNA could be important to repair UV-induced damage at yeast telomeres?

Answer: We acknowledge the interesting findings of the d’Adda di Fagagna’s lab but have no data to support this notion for UV induced DNA damage and its repair. Nevertheless, as requested by the Referee, we added a comment about DNA damage-induced lncRNA to the “Discussion”; P. 16, L. 425-431.
